# Machine Learning for Data Linkage

**DOI:** 10.23889/ijpds.v8i2.2240

**Published:** 2023-09-14

**Authors:** Rhosanna Ellum, Alex Lewis, Kristina Xhaferaj, Rachel Shipsey, Viktor Račinskij, Zoe White

**Affiliations:** 1 ONS, London, United Kingdom; 2 ONS, Titchfield, United Kingdom

Data linkage traditionally uses deterministic and probabilistic methods. Alternatively, machine learning methods can be applied as classification algorithms, using the data to inform decisions. This project compared the quality, in terms of precision and recall, of traditional methods with selected machine learning methods when applied to a standard linkage problem.

Two supervised methods, gradient boosted trees (GBT) and multiple layered perceptron classifier (MLPC), and one unsupervised method, maximum entropy classification (MEC), were implemented. The England and Wales 2021 Census to Census Coverage Survey (CCS) linkage was used as a gold-standard (GS) linked dataset to provide training samples for the supervised methods as well as testing samples for all methods. The F1 score (harmonic mean of precision and recall) was used to compare the performance of the models and to determine the optimal parameters and thresholds.

The Splink implementation of Fellegi-Sunter with Expectation Maximisation was used as a baseline for comparison.

The methods, trained on a sample of the GS, were used to link census and CCS data. All methods performed well with MEC achieving the highest precision (99.79%) but lowest recall (96.36%). The MLPC model achieved the highest F1 score (98.94%).

To understand the implications of not retraining supervised models for each dataset, the models were also used to link Census to a health dataset. The supervised models were not retrained using the health data; instead, the optimised GS models were applied. MEC had the lowest precision (96.51%) but the highest recall (98.48%) and highest F1 score (97.49%). With F1 scores of 96.99% and 96.14% respectively, the GBT and MLPC supervised models were not far behind in performance, despite not being trained using health data.

We have shown that machine learning methods can be used effectively for data linkage problems. Unsurprisingly, supervised models perform best when trained on and applied to the same data. Further research into generic training may allow us to use both supervised and unsupervised machine learning models for future data linkage.

